# Nanoscale Delivery of Resveratrol towards Enhancement of Supplements and Nutraceuticals

**DOI:** 10.3390/nu8030131

**Published:** 2016-03-02

**Authors:** Ana Rute Neves, Susana Martins, Marcela A. Segundo, Salette Reis

**Affiliations:** 1 UCIBIO, REQUIMTE, Department of Chemical Sciences, Faculty of Pharmacy, University of Porto, Rua de Jorge Viterbo Ferreira, 228, 4050-313 Porto, Portugal; rutepneves@gmail.com (A.R.N.); msegundo@ff.up.pt (M.A.S.); 2 Laboratory for Pharmaceutical Technology/Research Centre in Pharmaceutical Sciences, Faculty of Pharmacy, University of Porto, Rua de Jorge Viterbo Ferreira, 228, 4050-313 Porto, Portugal; susana.am.martins@gmail.com

**Keywords:** resveratrol, biocompatibility, oral bioavailability, nanodelivery systems, lipid nanoparticles, intestinal permeability

## Abstract

Resveratrol was investigated in terms of its stability, biocompatibility and intestinal permeability across Caco-2 cell monolayers in its free form or encapsulated in solid lipid nanoparticles (SLNs) and nanostructured lipid carriers (NLCs). SLNs and NLCs presented a mean diameter between 160 and 190 nm, high negative zeta potential of −30 mV and resveratrol entrapment efficiency of 80%, suggesting they are suitable for resveratrol oral delivery. Nanoencapsulation effectively protected resveratrol from photodegradation, and MTT assays demonstrated that neither resveratrol nor lipid nanoparticles adversely affected cell viability and integrity of Caco-2 cell monolayers. The *in vitro* intestinal permeability of resveratrol was significantly increased by NLCs, and SLNs did not impair the absorption of resveratrol. Resveratrol oral absorption can be enhanced during meals, since the intestinal permeability was increased in the presence of fed-state intestinal juices. SLNs and NLCs constitute carrier systems for resveratrol oral administration, for further use as supplements or nutraceuticals.

## 1. Introduction

Resveratrol has been one of the most extensively studied naturally occurring compounds due to its great therapeutic potential in cancer therapy, cardio- and neuroprotection, anti-inflammatory action, antioxidant activity, antiaging effects and diabetes treatment [1,2,3]. However, resveratrol pharmacokinetic properties must be considered since this compound demonstrated low solubility, rapid degradation and extensive metabolism, resulting in poor oral bioavailability [4,5,6,7]. Moreover, *trans*-resveratrol, the biologically active isomer, is highly photosensitive, being rapidly converted to *cis*-resveratrol when exposed to light [8]. Different strategies have been implemented to overcome the above-mentioned problems, such as the co-administration with metabolism inhibitors, use of analogs, and the design of new delivery systems [9,10,11,12,13,14]. In the past decade, several nanoformulations focused on enhancing therapeutic potential of resveratrol [3,9,15], namely polymeric nanoparticles such as poly(lactide) (PLA) nanoparticles [16,17], poly-(lactic-co-glycolic) acid (PLGA) [18,19,20] and poly(epsilon-caprolactone) (PCL) nanoparticles [18,19], polymeric micelles [21], nanoparticles based on zein, an amphiphilic protein [22], lecithin-based nanoemulsions [23], cyclodextrins [24,25,26], and liposomes [27,28,29,30,31], or on dual nanoencapsulation approaches, such as the inclusion of cyclodextrin complexes inside liposomes [32]. In the present study, we have developed resveratrol nanodelivery systems based on lipid nanoparticles to increase its oral bioavailability for further use as supplements or nutraceuticals. So far, lipid nanoparticles for resveratrol encapsulation have been developed for dermal and brain delivery [33,34,35], and only two studies have used it for oral administration [36,37]. Solid lipid nanoparticles (SLNs) and nanostructured lipid carriers (NLCs) have proven to be suitable vehicles for resveratrol encapsulation [36]. While SLNs are made solely by solid lipids at room and body temperature, NLCs are composed by a mixture of both solid and liquid lipids [38,39]. The incorporation of a liquid lipid to the solid matrix of the nanoparticles seems to create an imperfect matrix with an increased number of cavities, facilitating the accommodation of the encapsulated compound, while preventing early crystallization and drug release [40,41]. Therefore, resveratrol stability, biocompatibility, and intestinal permeability studies were conducted in a solution or encapsulated in SLNs and NLCs. Intestinal apparent permeability was assessed using Caco-2 cell monolayers grown on permeable filter transwell devices, simulating the intestinal membrane barrier. Caco-2 cell monolayers are the most commonly used intestinal models to evaluate the effect of delivery systems on drug permeation because Caco-2 cells form differentiated monolayers with microvilli, tight junctions, enzymes and transport systems, after 21 days of growth [42,43]. In addition, the influence of physiological fluids that mimic gastrointestinal conditions on the apparent permeability of resveratrol and resveratrol-loaded nanoparticles was also tested. Fasted-state simulated intestinal fluid (FaSSIF) and fed-state simulated intestinal fluid (FeSSIF), besides the control medium Hanks’ balanced salt solution (HBSS), were used. The first two media mimic fasted and fed digestive conditions because they contain natural surfactants (bile salts and lecithin) that form micelles that are present in the gastrointestinal fluids [44,45].

## 2. Materials and Methods

### 2.1. Materials

*trans*-Resveratrol (>99% purity) was purchased from Sigma-Aldrich (St. Louis, MO, USA), cetyl palmitate was provided by Gattefossé SAS (Nanterre, France), polysorbate 60 (tween 60) was supplied by Merck (Darmstadt, Germany), and miglyol-812 from Acofarma (Madrid, Spain). For the preparation of buffer solutions, sodium hydroxide was obtained from Riedel-de Haën AG (Seelze, Germany), sodium phosphate monobasic monohydrated was acquired from Fluka (Seelze, Germany), sodium chloride was purchased from Panreac (Barcelona, Spain), and acetic acid (≥99.8%) was obtained from Sigma-Aldrich. The transport media HBSS was purchased from Gibco (Paisley, UK), while FaSSIF and FeSSIF were prepared by using simulated intestinal fluid (SIF) instant powder (Phares Drug Delivery AG, Muttenz, Switzerland) according to the manufacturer’s instructions. The HPLC mobile phase was composed of acetonitrile and acetic acid (LiChrosolv HPLC grade) obtained from Merck. Caco-2 cell line was purchased from the American Type Culture Collection (ATCC, Wesel, Germany) between passage numbers 35 and 55, and maintained in Dulbecco’s Modified Eagle Medium (DMEM) supplemented with 10% fetal bovine serum (South America origin), 1% fungizone (amphotericin B, 250 μg·mL^−1^), and 1% Pen Strep (penicillin, streptomycin), all obtained from Gibco. Trypsin-EDTA was also purchased from Gibco. Trypan blue and thiazolyl blue tetrazolium bromide (MTT) were both provided by Sigma-Aldrich.

### 2.2. Preparation of SLNs and NLCs

SLNs and NLCs were produced by a high shear homogenization technique followed by the ultrasound method to reduce the microparticles to the nanometer range, as described before [36,46]. SLNs were composed of 10% of the solid lipid cetyl palmitate, while NLCs were constituted by 7% of cetyl palmitate and 3% of the liquid lipid miglyol-812. Both types of lipid nanoparticles were dispersed in aqueous medium by the presence of 2% surfactant polysorbate 60. Resveratrol (5 mg) was added to the lipid phase and melted above the lipid’s melting point, which was followed by dispersion in the aqueous phase. The oil/water (o/w) emulsion was then submitted to high-speed stirring (SLNs—30 s at 12,000 rpm and NLCs—2 min at 12,000 rpm) in Ultra-Turrax T25 (Janke and Kunkel IKA-Labortechnik, Staufen, Germany) and sonicated (SLNs—5 min at 80% intensity and NLCs—15 min at 70% intensity) in a Sonics and Materials Vibra-Cell™ CV18 (Newtown, CT, USA). After cooling at room temperature, lipid nanoparticles were formed and the formulations were stored for characterization and application.

### 2.3. Resveratrol Entrapment Efficiency

Resveratrol entrapment efficiency was measured indirectly by quantifying the amount of compound that was not internalized by the nanoscale particles, as dissolved compound in the aqueous phase of the formulation [38,47]. Amicon Ultra-4 centrifugal Filter Devices (Millipore, Billerica, MA, USA) were used to separate lipid nanoparticles from the aqueous medium after 15 min of 3300 *g* centrifugation in a Jouan BR4i multifunction centrifuge (Thermo Electron, Waltham, MA, USA). The non-entrapped resveratrol was quantified in the supernatant after absorbance spectral analysis using a V-660 spectrophotometer (Jasco, Easton, MD, USA) at 200–600 nm and the entrapment efficiency calculated according to the equation (1):

(1) $$\text{EE}=\frac{\text{total resveratrol}-\text{non entrapped resveratrol}}{\text{total resveratrol}}\times 100$$

### 2.4. Particle Size and Zeta Potential Measurements

Particle size and zeta potential analysis were performed by dynamic light scattering (DLS) and electrophoretic light scattering (ELS), respectively, in a Brookhaven Instrument (Holtsville, NY, USA). The samples were first diluted (1:400) in order to get a suitable scattering intensity. The mean hydrodynamic diameter (Z-average) and the polydispersity index were determined as a measure of the width of particles size distribution, while zeta potential was measured according to the electrophoretic mobility of nanoparticles, which is influenced by their charge. The measurements were performed in triplicate in cycles of 10 runs each.

### 2.5. Resveratrol Photostability Study

Photostability studies were performed to investigate the protection effect of lipid nanoparticles against photodegradation of *trans*-resveratrol over time. Aqueous solutions of free resveratrol and resveratrol encapsulated in SLNs and NLCs at the same concentration of 1 mg·mL^−1^ were exposed for 4 h to UV light (HPW125W-T E27, Philips) in a chamber with reflecting walls. The UV lamp had an emission spectrum between 320 and 400 nm, with an irradiation intensity of 5.5 × 10^−3^ kJ·s^−1^·m^−2^. Aliquots were withdrawn from each sample at 0, 0.5, 1, 2, 3 and 4 h, and a 20%-volume of ACN was added to promote the disruption of lipid matrix, allowing the quantification of *trans*-resveratrol in a solution by spectrophotometric detection at 306 nm in a V-660 spectrophotometer (Jasco, Easton, MD, USA). Resveratrol photodegradation was calculated as a percentage by comparing the absorbance of *trans*-resveratrol at each time to the maximum absorbance at the beginning of each experiment.

### 2.6. Caco-2 Cell Culture

Caco-2 cells were grown in DMEM supplemented with 10% fetal bovine serum, 1% fungizone, 1% Pen Strep at 37 °C, and 5% CO_2_. Cells were supplied with a fresh medium every 2 days and subcultured by treatment with 0.25% trypsin–EDTA when they reached 80%–90% confluency, followed by counting in a Neubauer chamber with a trypan blue solution (0.4%, w/v). Cells were then seeded at a density of 10^4^ cells per cm^2^.

### 2.7. MTT Cell Viability Assay

Cell viability in the presence of nanoparticles was assessed by MTT assay. This assay detects living cells via mitochondrial dehydrogenases activity [48]. Cells were seeded in 96-well plates (10^4^ cells per well) and grown for 20 h at 37 °C, 5% CO_2_. Cells were then incubated with different concentrations of free resveratrol and resveratrol-loaded SLNs and NLCs, as well as with the respective placebo formulations for 4 h. After that, cells were treated with 0.5 mg/ml MTT for 4 h. Finally, DMSO was added to dissolve MTT formazan and incubated for 15 min, followed by absorbance measurement at 550 and 690 nm in a microplate reader (Synergy HT; Bio-Tek Instruments, Winooski, VT, USA).

### 2.8. Caco-2 Cell Permeability Study

Cells were seeded on transwell polycarbonate inserts (12 wells, pore diameter of 3 µm, 1.12 cm^2^) in a density of 1 × 10^5^ cells per insert and grown for 21 days, at 37 °C and 5% CO_2_, until a confluent monolayer was achieved. In fact, Caco-2 cells present an absorptive polarized monolayer and an apical brush border after 21 days from seeding [49,50]. The integrity of the monolayers was monitored by measuring trans-epithelial electrical resistance (TEER) with an epithelial voltohmmeter (EVOM) from World Precision Instruments (Sarasota, FL, USA). Only monolayers with TEER values higher than 200 Ω cm^2^ were used. Permeability assay was performed by incubating free-resveratrol and resveratrol-loaded SLNs and NLCs (10 μM) on the apical side for 4 h at 37 °C and 5% CO_2_. Three different transport media were used to mimic intestinal fluids: HBSS was used as control, FaSSIF as the fasted state medium, and FeSSIF as the fed state medium. Aliquots from the basolateral side were collected at regular intervals of 30 min and treated with 20% acetonitrile/acetic acid (92:8, v/v) to promote the disruption of lipid nanoparticles and the release of resveratrol to be quantified. The quantification was performed by HPLC separation in a C18 monolithic column with isocratic elution in a 2% acetic acid solution/acetonitrile (80:20), followed by fluorimetric detection (330/374 nm), according to a validated method [51]. TEER values were rechecked at the end to confirm the quality of the monolayer after the experiment. The apparent permeability coefficients (P_app_) in the three media tested were calculated after 4-h assay by the Equation (2):

(2) $$P_{app}\left(cm/s\right)=\;\frac{Q}{A\;\times \;C\;\times \;t}$$

where *Q* represents the total amount of permeated resveratrol (μg), *A* is the surface area of the insert (cm^2^), *C* is the initial resveratrol concentration (μg/cm^3^) and *t* is the experiment time (s).

### 2.9. Statistical Analysis

Statistical analysis was performed using SPSS software (version 20.0; IBM, Armonk, NY, USA). The measurements were repeated at least three times and data were expressed as mean ± SD. Data were analyzed using one-way analysis of variance (one-way ANOVA), followed by Bonferroni, Tukey and Dunnett *post-hoc* tests. A *p* value less than 0.05 was considered statistically significant.

## 3. Results

### 3.1. Characterization of Nanoparticles

The physicochemical characterization of nanoparticles is depicted in Table 1. All formulations presented a size between 160 and 190 nm, polydispersity index of 0.2 and high negative zeta potential around −30 mV, regardless of resveratrol incorporation. The results indicate that there is no statistically significant difference in the size and charge of both types of lipid nanoparticles (SLNs and NLCs) and that resveratrol did not significantly change these parameters. Resveratrol entrapment efficiency in both SLNs and NLCs was found to be very high (around 80%), suggesting its preferential partition into the nanoparticles lipid matrix. The developed nanoparticles can be considered physically stable because the absolute value of zeta potential is around 30 mV, and the electrostatic repulsions between particles can therefore avoid flocculation and aggregation of nanoparticles [52,53]. Moreover, polydispersity index of 0.2 suggests an acceptable monodispersity distribution, with low variability. The mean size of these nanosystems (<200 nm) confirmed that they are appropriated for oral administration and gastrointestinal absorption [54,55], and the negatively charged nanoparticles can interact with enterocytes, being prone for permeation across the intestinal barrier [56,57].

### 3.2. Photostability Study of Resveratrol

Resveratrol is found in nature as both *trans* and *cis* isomers, *trans*-resveratrol being the biologically active form [58]. However, it is well-known that *trans*-resveratrol easily isomerizes into the *cis* form under UV irradiation [8,59].

**Figure 1:**
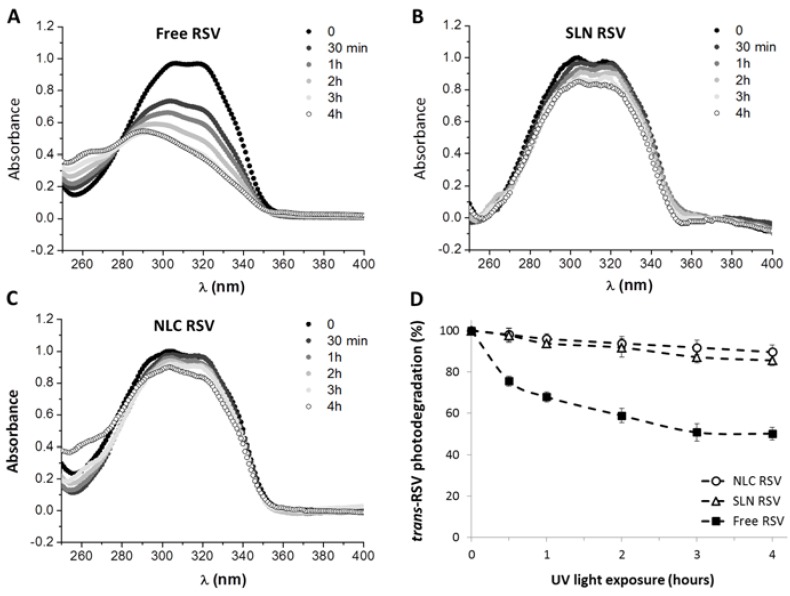
Photostability study of *trans*-resveratrol exposed to 312 nm UV light for four hours. (**A**) free resveratrol; (**B**) resveratrol loaded in SLNs; (**C**) resveratrol loaded in NLCs; (**D**) Photodegradation profile of *trans*-resveratrol in aqueous solution (■) compared to the compound encapsulated in SLNs (∆) and NLCs (○).

Figure 1A displays the absorption intensity spectra of resveratrol before (*trans*-resveratrol) and after UV light exposure during 4 h. The result is in agreement with previous studies which show a decrease of 50% in the *trans*-resveratrol absorption intensity at 306 nm and a 20 nm shift from 306 nm to 286 nm for the maximum absorption intensity, typical of *cis*-resveratrol spectra [60,61]. The ability of lipid nanoparticles to protect the compound against isomerization was also investigated and the absorption spectra for resveratrol-loaded SLNs and NLCs before and after UV irradiation are represented in Figure 1B and Figure 1C, respectively. Resveratrol photostability after 4 h of UV light exposure was verified by comparing the percentage of photodegradation inside lipid nanoparticles with that of the pure compound in an aqueous solution at the same concentration. The results suggest that both types of lipid nanoparticles (SLNs and NLCs) enhance photostability of resveratrol (Figure 1D). In fact, around 50% of *trans*-resveratrol in solution was converted to the *cis* isomer after 4h of UV exposure and the entrapment of the compound inside lipid nanoparticles decreased this value for less than 10%. This result is consistent with previous studies using either SLNs or liposome delivery systems [28,37,62].

### 3.3. Caco-2 Cell Viability Study

**Figure 2:**
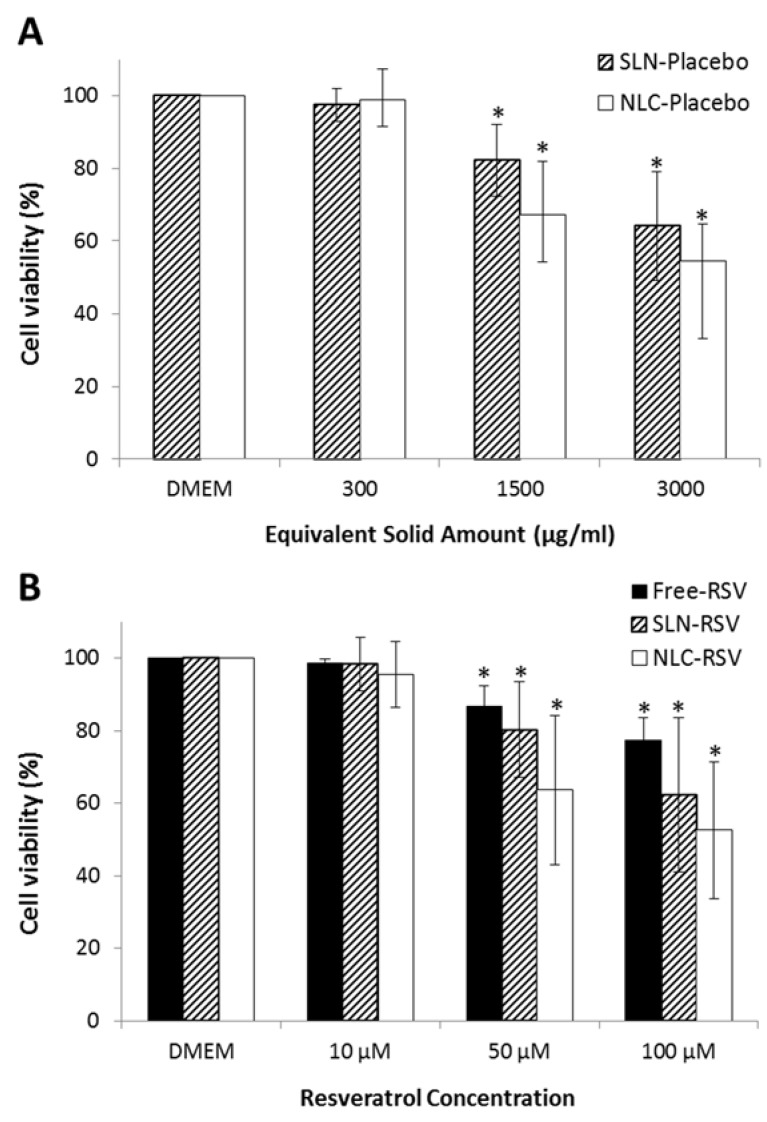
Caco-2 cell viability assessed by MTT assay after 4 h of incubation with increasing concentrations of samples. (**A**) Placebo SLNs (▨) and placebo NLCs (□) formulations; (**B**) free resveratrol (■) and resveratrol-loaded SLNs (▨) or NLCs (□). **Note:** All values represent the mean ± standard deviation (*n* = 3). Results were analyzed and compared with a DMEM medium, which represents the maximum of cell viability. (*) denotes statistically significant differences (*p* < 0.05) from DMEM.

Caco-2 cell viability studies were performed to assess the cytotoxicity of resveratrol and resveratrol-loaded lipid nanoparticles in the intestinal barrier. MTT assay evaluates the activity of cellular oxidoreductase enzymes inside mitochondria by converting the MTT tetrazolium dye into its insoluble formazan, which has a purple color. The quantitative measure of purple that is produced (550 nm) is directly proportional to the number of viable cells, allowing the determination of the biocompatibility of formulations. Observing Figure 2, it is possible to conclude that 10 μM concentration of resveratrol, resveratrol-loaded nanoparticles and the equivalent solid amount of placebo nanoparticles did not produce any cytotoxic effect after 4 h of incubation at 37 °C, when compared to cells in DMEM medium. Only at concentrations higher than 50 μM was there a significant reduction of cell metabolic activity. However, even for the highest concentration tested (100 μM), a cell toxicity below 50% was produced, indicating that resveratrol-loaded nanoparticles are biocompatible and well tolerated by the intestinal mucosa. This study also allowed us to choose the appropriate, non-toxic concentration (10 μM) for the intestinal permeability studies, without compromising Caco-2 cell monolayer.

### 3.4. Intestinal Permeability Study

**Figure 3:**
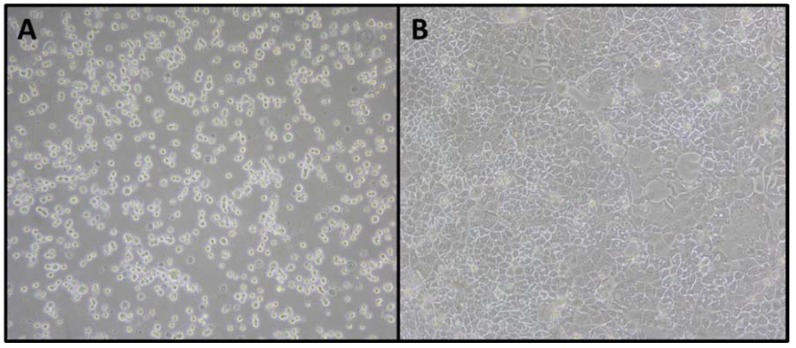
Unstained photographs of Caco-2 cells. (**A**) Immediately after seeding and (**B**) with 100% of confluence. Magnification: 100×.

*In vitro* intestinal permeability assays were performed in transwell devices using Caco-2 cell monolayers that mimic the intestinal barrier. Figure 3 shows Caco-2 cells photographs immediately after seeding and after forming a confluent monolayer. Confluent Caco-2 cells have been used as the standard model for the *in vitro* investigation of intestinal absorption due to their similarities to the small intestinal epithelium [63,64].

**Figure 4:**
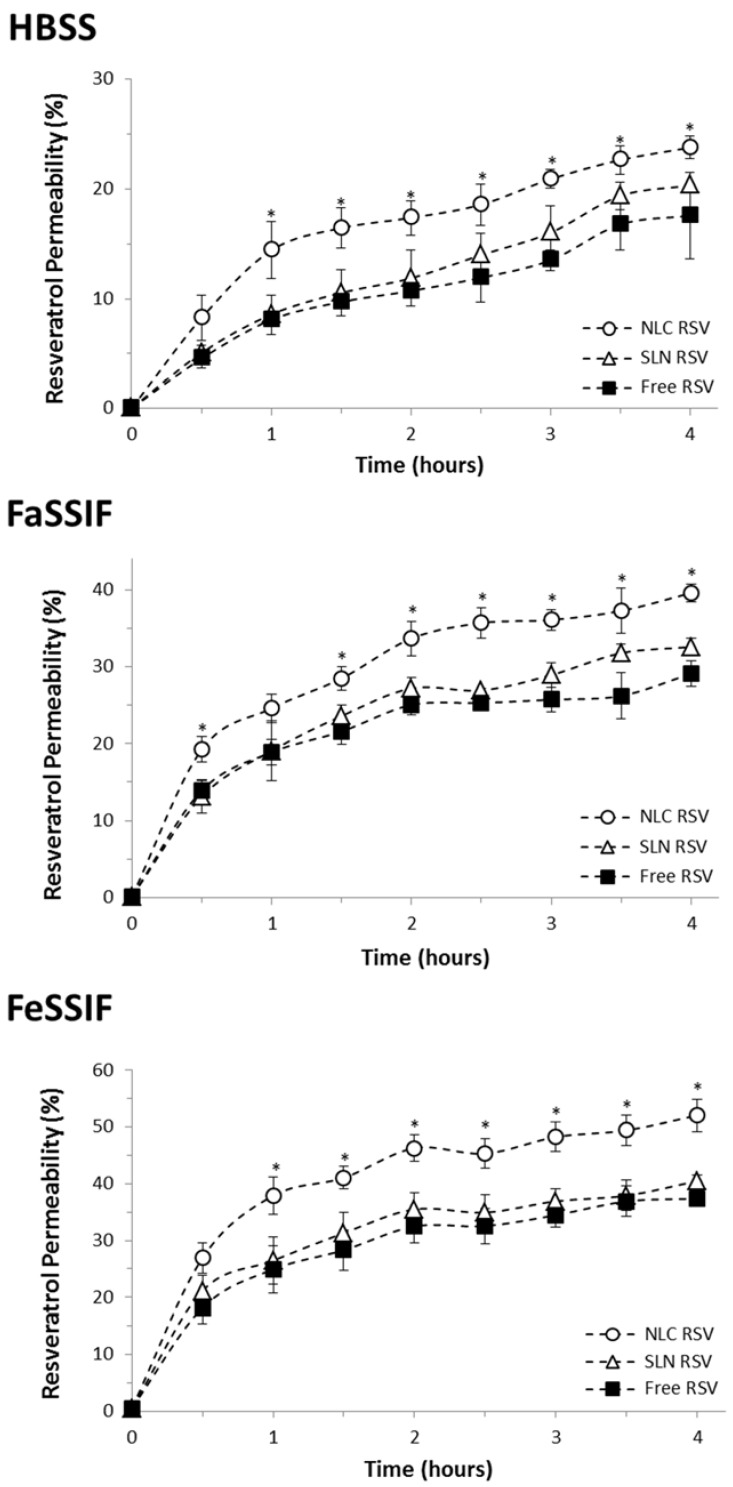
Resveratrol permeability over 4 hours of cumulative transport across Caco-2 cell monolayer mimicking intestinal permeability conditions, on free-form (■) and encapsulated in SLNs (∆) or NLCs (○) in 3 different transport media. (**A**) HBSS; (**B**) FaSSIF and (**C**) FeSSIF. Note: All values represent the mean ± standard deviation (*n* = 3). Results were analyzed and compared with the free form of resveratrol. (*) denotes statistically significant differences (*p* < 0.05).

In order to mimic the intestinal fluids, three transport media were applied: HBSS, FaSSIF and FeSSIF. HBSS was used as the control medium, while the last two media mimic the fasted- and fed-state intestinal juices, respectively, because they contain natural surfactants (bile salts and lecithin) that generate micelles that are present during the digestive process [44,45]. The HPLC quantification method was firstly developed and validated for resveratrol in the three different matrices [51]. The permeation of free resveratrol and resveratrol-loaded lipid nanoparticles across the intestinal barrier is presented as cumulative transport over 4 h in Figure 4. The results are also reported as P_app_ (Table 2), which provides independent values that can be compared between different studies. Figure 4 shows that resveratrol rapidly starts to cross Caco-2 monolayer, since the compound is detected on the basolateral side after only 30 min. This initial burst permeability has already been reported in previous studies where P_app_ of free resveratrol is in agreement with our value of 1.6 × 10^−5^ cm/s in HBSS medium [65,66]. The comparison between different transport media indicates that resveratrol P_app_ is 1.7-fold higher in FaSSIF and 2.1-fold higher in FeSSIF when compared to HBSS control medium. This fact can be related to the micellarization of resveratrol and lipid nanoparticles [45]. On the other hand, FaSSIF and FeSSIF protection against *trans*-resveratrol degradation is also reported [51], which can explain the increased resveratrol absorption when taken with meals. We have performed identical studies to ensure the integrity and stability of the nanoparticles in the different media used, in order to ensure the validity of the intestinal permeability assay. At regular intervals, when aliquots were collected from the basolateral side of Caco-2 monolayer, the size and zeta potential parameters of these aliquots were analyzed, and no statistically significant changes were detected compared to the size and zeta potential values of the fresh nanoparticles reported before (Table 1). This information indicates that the permeability results are justified by the permeation of the SLNs and NLCs themselves through the intestinal barrier and not to the diffusion of the free compound.

When comparing the different formulations tested, resveratrol permeability through Caco-2 monolayers was significantly increased by NLCs encapsulation (up to 1.5-fold increment, *p* < 0.05). On the other hand, SLNs did not produce any significant effect on resveratrol permeation. The difference between both types of lipid nanoparticles may be related with their different lipid composition, because their size, charge and resveratrol entrapment efficiency were found to be similar. In a recent study, we have found that NLCs promoted a more sustained and controlled release of resveratrol when compared to SLNs in gastrointestinal studies, since SLNs release 10% of resveratrol after 4 h assay, while NLCs only release 5% of compound at the same conditions [36]. The difference observed can be attributed to the less ordered lipid matrix conferred by the liquid lipid present in the NLCs, allowing better accommodation of resveratrol and avoiding its premature release. Therefore, although the encapsulation efficiency was identical in both systems, the early loss of compound in SLNs may have led to a lower permeability across the intestinal barrier when compared to NLCs. Besides that, the presence of a liquid lipid in NLCs composition can influence the lipid matrix of those nanoparticles that become more flexible, thus allowing a greater physical deformation at the time of crossing the barrier, and perhaps leading to an increase of resveratrol permeation. Indeed, the higher permeability of NLCs could be related to the decrease of tight junctions stickiness, promoting an enhancement of the paracellular transport route with this particular system. Actually, TEER values in the presence of NLCs were slightly smaller (10%) than those for SLNs and free-resveratrol, indicating a partial opening of tight junctions between epithelial cells when NLCs were incubated. Nevertheless, the TEER values were reverted at the end of the assay by replacing the nanoparticles solution by fresh medium, indicating that the opening of tight junctions was reversible and did not completely disrupt and compromise the monolayer integrity [67]. These results are in agreement with a recent paper where the applicability of SLNs and NLCs as oral drug delivery nanosystems was tested [68]. In this study, the cellular uptake, internalization pathways and transcytosis routes through Caco-2 monolayers were assessed. Transmission electron microscopy and confocal laser scanning microscopy images revealed the ability of these nanosystems to cross the monolayer by a transcellular route. Indeed, cell internalization occurred mainly through a clathrin-mediated endocytosis mechanism. However, the higher cellular uptake of NLCs together with the contribution of a paracellular route also involved in this nanocarrier permeation would explain the higher permeability found for NLCs compared to SLNs. These results support the conclusions drawn in the present study.

Therefore, we conclude that NLCs exhibit greater efficiency in the transport across the intestinal barrier, promoting an increase in resveratrol permeability. Regarding SLNs, they do not produce any significant increase in resveratrol permeation, but still do not compromise its absorption when compared with the free compound. Thus, SLNs may also be used for resveratrol delivery into systemic circulation, since they also provide gastrointestinal protection and resistance to the early first-pass metabolism [37]. As a conclusion, both nanosystems might be used for oral administration of resveratrol.

## 4. Discussion

In the present study, lipid nanoparticles were successfully developed, characterized and applied for oral administration of resveratrol. SLNs and NLCs presented a mean diameter of 160–190 nm, high negative zeta potential of −30 mV and a resveratrol entrapment efficiency around 80%, suggesting that they are suitable for resveratrol oral delivery, being prone for its permeation across the intestinal barrier [55,57]. The results also revealed that resveratrol encapsulation effectively protected it from photodegradation, and previous studies have already shown that these nanosystems conferred protection during its gastrointestinal transit avoiding premature release in simulated gastric and intestinal conditions [36]. Therefore, this study demonstrated the applicability of these nanoparticles, protecting resveratrol from degradation as well as allowing their permeability through the intestinal barrier in order to reach the systemic circulation. Indeed, SLNs did not impair the absorption of resveratrol, and NLCs further increased the permeability of the polyphenol compound across Caco-2 cell monolayer. Furthermore, it was observed that resveratrol oral absorption can be enhanced during meals, since the intestinal permeability was increased when fed-state intestinal juices were applied. At this point, the development of resveratrol-loaded nanoparticles may be important towards the production of novel food supplements or nutraceuticals. This can be achieved by improving products derived from food sources, such as juices, yogurts, milk, or cheese, with extra health benefits similar to those attributed exclusively to resveratrol-rich red wine consumption. A combination of several innovative formulation strategies have been applied in order to accomplish this great challenge [3,9,15], including the use of polymeric nanoparticles [16,17,18,19,20], polymeric micelles [21], zein-based nanoparticles [22], lecithin-based nanoemulsions [23], cyclodextrins [24,25,26], liposomes [27,28,29,30,31,32], and lipid nanoparticles [33,34,35,36,37]. The efforts in tuning these intelligent nanocarriers are based on improving the permeation across an epithelial cell monolayer, on preserving the physical integrity of the nanoparticles during the biodistribution process, and on enhancing the activity of the encapsulated resveratrol. Here, lipid nanoparticles not only meet these physical, chemical and biological requirements, as exploited before, but also represent the most suitable nanosystems for food industry application. In fact, SLNs and NLCs are composed of biodegradable and biocompatible lipids recognized as safe by Food and Drug Administration, which can be loaded with the lipophilic and poorly soluble resveratrol, for further use in food supplements [38,47]. Moreover, the preparation of lipid nanoparticles is very simple, rapid and economically affordable for food industry scale up [52].

## 5. Conclusions

As a conclusion, SLNs and NLCs were developed and validated for *trans*-resveratrol protection, stabilization and intestinal permeability. Both lipid nanoparticles seem to be compatible and adequate for resveratrol delivery after oral administration, representing a promising strategy for enhancing its *in vivo* efficacy.

## Figures and Tables

**Table 1:** Characterization of resveratrol-loaded solid lipid nanoparticles (SLNs) and nanostructured lipid carriers (NLCs).

	Z-Average (nm)	Polydispersity Index	Zeta Potential (mV)	Entrapment Efficiency (%)
SLN Placebo	189.2 ± 15.4	0.205 ± 0.045	−30.8 ± 7.3	-
SLN RSV	171.5 ± 17.1	0.215 ± 0.033	−32.1 ± 6.9	80.5 ± 3.4
NLC Placebo	172.9 ± 19.8	0.203 ± 0.030	−29.6 ± 7.4	-
NLC RSV	163.8 ± 21.7	0.198 ± 0.027	−29.9 ± 5.8	78.9 ± 2.5

**Note:** All values represent the mean ± standard deviation (*n* = 3).

**Table 2:** Apparent permeability (P_app_) of free resveratrol and resveratrol-loaded SLN or NLC for 4 h of transport across Caco-2 cell monolayer mimicking intestinal permeability conditions.

	P_app_ (×10^−5^ cm/s)		
	HBSS	FaSSIF	FeSSIF
NLC RSV	2.2 ± 0.1 *	3.7 ± 0.1 *	4.8 ± 0.3 *
SLN RSV	1.9 ± 0.2	3.0 ± 0.1	3.8 ± 0.1
Free RSV	1.6 ± 0.4	2.7 ± 0.2	3.5 ± 0.4

Note: All values represent the mean ± standard deviation (*n* = 3). (*) denotes statistically significant differences compared with the free form of resveratrol (*p* < 0.05).
